# The Chemokine (C-C Motif) Receptor 1 Antagonist BX471 Improves Fluid Resuscitation in Rat Models of Hemorrhagic Shock

**DOI:** 10.3390/biomedicines13051241

**Published:** 2025-05-20

**Authors:** Elizabeth A. Cook, Ololade Ogunsina, Xianlong Gao, Matthias Majetschak

**Affiliations:** 1 Department of Surgery, Morsani College of Medicine, University of South Florida, Tampa, FL 33612, USA; eac1@usf.edu (E.A.C.); oogunsina@usf.edu (O.O.); xgao1@usf.edu (X.G.); 2 Department of Molecular Pharmacology & Physiology, University of South Florida, Tampa, FL 33612, USA

**Keywords:** chemokine receptor antagonists, chemokine (C-C motif) receptor 1, hemorrhagic shock, fluid resuscitation strategies

## Abstract

**Background/Objectives**: We reported previously that antagonists at chemokine receptors CCR2 and CCR3 have fluid-sparing effects during resuscitation from hemorrhagic shock. Because CCR1 shares several chemokine ligands with CCR2/3, we tested whether the CCR1 antagonist BX471 also reduces fluid requirements to maintain hemodynamics. **Methods**: Sprague Dawley rats were hemorrhaged for 30 min, followed by fluid resuscitation to maintain blood pressure for 60 min (series 1) and 180 min (series 2). Series 1: Animals received vehicle (n = 5), 0.05 μmol/kg (n = 5), or 0.5 μmol/kg (n = 4) BX471 at t = 30 min. Series 2: Animals received vehicle (n = 8) or 0.5 μmol/kg (n = 7) BX471 at t = 30 min. Hemodynamics, fluid requirements, blood gases, and lactate were monitored. Serum concentrations of CCR1 ligands (CCL3/4/5/7) were determined at baseline and at the conclusion of the experiments. Tissue (small/large intestine, lung) wet/dry (W/D) weight ratios, lung myeloperoxidase activity, and a panel of inflammation markers in tissue extracts were measured. **Results**: All animals could be resuscitated to target blood pressures. Series 1: A total of 0.5 μmol/kg BX471 reduced fluid requirements by more than 60% (*p* < 0.05 vs. vehicle and 0.05 μmol/kg BX471). Series 2: Systemic CCL3/5/7 levels increased during the experiment (*p* < 0.05). BX471-treatment reduced fluid requirements by more than 60% (*p* < 0.05) and prevented increases in CCL3/7. W/D ratios of large intestine and of the sum of all tissues were lower with BX471 treatment (*p* < 0.05). BX471-treatment reduced TNFα and IL6 concentrations in large intestine extracts (*p* < 0.05). **Conclusions**: Our findings suggest CCR1 as a new therapeutic target to reduce fluid requirements during resuscitation from hemorrhagic shock.

## 1. Introduction

Chemokines and their receptors play critical roles as mediators of inflammation. Numerous chemokine receptor antagonists have been developed and explored as potential therapeutics for inflammatory disease processes [1].

Chemokine (C-C motif) receptor 1 (CCR1) is expressed in numerous cell types and tissues, including leukocytes, endothelial cells and vascular smooth muscle cells, and is thought to play important roles in the pathogenesis of multiple disease processes, including but not limited to rheumatoid arthritis, multiple sclerosis, eosinophilic airway inflammation, and multiple myeloma [2,3,4,5,6,7]. Potent CCR1 antagonists have been developed, and several have entered clinical trials, including BX471, which was tested for therapeutic potential in patients with multiple sclerosis [8,9]. Although BX471 failed to show therapeutic efficacy for multiple sclerosis, a 16-week dosing of BX471 was well tolerated [9]. BX471 is an orally active non-peptide CCR1 antagonist with high selectivity [10], and beneficial effects of BX471 have been reported in several preclinical models, such as pancreatitis, sepsis or allograft rejection models [11,12,13].

Recently, we tested the effects of CCR2, CCR3, and CCR5 antagonists in rat models of hemorrhagic shock and subsequent fluid resuscitation [14,15]. While the CCR5 antagonist Maraviroc did not affect fluid resuscitation requirements, we observed fluid-sparing effects of the CCR2 antagonist INCB3284 and the CCR3 antagonist SB328437. Although the significant fluid-sparing effects of SB328437 were limited to very short resuscitation periods after hemorrhagic shock, INCB3284 showed significant beneficial effects during resuscitation for up to 4 h after hemorrhagic shock, and dosing optimization improved its therapeutic efficacy [14,15]. These observations imply that activation of CCR2 and CCR3 contribute to the development of increased resuscitation fluid requirements after hemorrhagic shock.

Chemokines and their receptors are well known to be promiscuous in their receptor and ligand specificity, respectively [2,16,17,18]. CCR1 is evolutionary closely related to CCR2 and CCR3 [19,20], and shares multiple chemokine ligands, such as chemokine (C-C motif) ligand 5 (CCL5), CCL7, CCL8, CCL13, or CCL16, with CCR2 and CCR3 in humans and rodents [2]. Thus, it appears possible that the activation of CCR1 also affects resuscitation fluid requirements after hemorrhage, and that the blockade of CCR1 may possess therapeutic potential in pre-clinical models of hemorrhagic shock. The effects of the CCR1 antagonist BX471 in the acute setting of hemorrhagic shock and subsequent fluid resuscitation, however, have not been studied. Thus, the aim of this study was to evaluate the possible therapeutic potential of BX471 during resuscitation from hemorrhagic shock in rat models.

## 2. Materials and Methods

Drugs. BX471 was purchased from Tocris, Bio-Techne Corporation (Minneapolis, MN, USA).

Hemorrhagic Shock Models. All procedures were performed in accordance with the National Institutes of Health Guidelines for Use of Laboratory Animals and were approved by the Institutional Animal Care and Use Committee of the University of South Florida (IS00008139).

Male Sprague Dawley rats (320–400 g) were obtained from Envigo (Indianapolis, IN, USA). General anesthesia was first induced utilizing an isoflurane-soaked gauze and a bell jar. Once anesthetized, the animal was transferred to the procedural area, where sedation was maintained at 2.6% isoflurane utilizing nose cone inhalation with the SomnoSuite small animal anesthesia system (Kent Scientific Corporation, Torrington, CT, USA). This achieved a level of sedation such that the animal no longer responded to noxious stimuli but still maintained spontaneous respiration. Utilizing a direct cut-down method, the left femoral artery and right femoral vein were isolated and cannulated with a 24-gauge and 26-gauge catheter, respectively. The arterial line was used for hemodynamic monitoring and blood withdrawal, and the venous line was used for drug and fluid administration. Following cannulation, isoflurane was reduced to 1.7% and the animals were monitored for a 10 min period prior to starting the experiment to ensure hemodynamic and respiratory stability. Hemodynamics were monitored continuously with the Surgivet invasive blood pressure monitor (Med-Electronics, Beltsville, MD, USA). Two series were completed, with experiments performed in an alternating fashion. In both series, hemodynamics were monitored continuously and recorded at 2 min intervals. The level of sedation was maintained until the animal expired or was euthanized at the conclusion of the experiment.

*Series 1*: Rats were hemorrhaged to a mean arterial blood pressure (MAP) of 30 mmHg for 30 min. At the end of this shock period (t = 30 min), animals received either 1 mL of lactated ringer’s solution (LR) (=control group, n = 5), 1 mL of 0.05 µmol/kg BX471 (n = 5), or 1 mL of 0.5 µmol/kg BX471 (n = 4) in phosphate-buffered saline (PBS) as a bolus injection. The doses of BX471 in PBS were prepared from a stock solution of 10 mM BX471 dissolved in dimethyl sulfoxide (DMSO). The final DMSO concentrations in the BX471 preparations were between 0.15 and 1.5% *v*/*v*, which do not produce noticeable effects in numerous species, including rats [21]. The doses of BX471 were chosen based on the reported volume of the distribution of BX471 of 0.5 L/kg to result in systemic concentrations that equal or 10-fold exceed its Ki to inhibit chemokine binding to rat CCR1 of 121 ± 60 nM [10].

Thereafter, all animals received 1 mL boluses of LR until either systolic blood pressure (SBP) recovered to 90 mmHg or MAP recovered to 60 mmHg. Subsequent 0.5–1 mL boluses of LR were administered as needed to maintain this blood pressure goal for the entirety of the 60 min resuscitation period. Blood samples for the analyses of blood gases and laboratory parameters were collected at the beginning of hemorrhage (t = 0 min), the end of hemorrhage (t = 30 min), and at the end of fluid resuscitation (t = 90 min). At the conclusion of the experiment (t = 90 min), surviving animals were euthanized (5% isoflurane, bilateral pneumothorax, and ventriculotomy).

*Series 2*: Rats were hemorrhaged and resuscitated as in series 1 for 180 min. At the end of the shock period (t = 30 min), animals received 1 mL of LR (=control, n = 8) or 1 mL of 0.5 µmol/kg BX471 (n = 7) in PBS as a bolus injection. Blood samples were collected at the beginning of hemorrhage (t = 0 min), the end of hemorrhage (t = 30 min), at the midpoint (t = 120 min), and at the end of fluid resuscitation (t = 210 min) for the analyses of blood gases and laboratory parameters. Serum samples were collected at t = 0 min and t = 210 min and stored at −80 °C for subsequent analyses. At the conclusion of the experiment (t = 210 min), surviving animals were euthanized (5% isoflurane, bilateral pneumothorax, and ventriculotomy). Tissue samples of lung, small intestine, and large intestine were collected and used for the calculation of wet-weight to dry-weight (W/D) ratios or stored at −80 °C for further analyses.

Arterial Blood Gases and Laboratory Parameters: Arterial blood gases, electrolytes, creatinine, lactate, hemoglobin, and hematocrit were analyzed in both series at the time points outlined above with the use of the Element point of care veterinary blood gas, electrolyte, and critical care analyzer (Cuattro Veterinary USA, Loveland, CO, USA).

Tissue Extracts: Tissue samples (small and large intestine, lung) were weighed and placed in cold PBS at a 1:4 weight to volume ratio. Samples were homogenized with the Biospec Products Tissue-Tearor (Bartlesville, OK, USA) over four 15 s intervals with a 15 s of rest in between. The homogenates were then centrifuged at 15,000× *g* for 20 min, and the supernatants (=tissue extracts) were aliquoted and used for further analyses.

Protein Measurements: The colorimetric Bio-Rad Protein Assay Kit (Hercules, CA, USA) was used to measure total protein concentrations in serum samples and tissue homogenates according to the manufacturer’s instructions. Bovine serum albumin was used as the protein standard.

Measurements of Chemokine Concentrations: Serum levels of CCL3, CCL4, CCL5, and CCL7 were measured with commercially available rat enzyme-linked immunosorbent assays (ELISA, Boster Bio, Pleasanton, CA, USA) according to the manufacturer’s instructions. Chemokine concentrations were calculated per mg of total protein.

Measurements of Myeloperoxidase (MPO) Activity: Relative myeloperoxidase (MPO) activity was measured in tissue homogenates utilizing the commercially available fluorescent EnzChek Myeloperoxidase (MPO) Activity Assay Kit according to the manufacturer’s instructions (Thermo Fisher Scientific, Waltham, MA, USA). MPO activity was calculated as relative fluorescence units (RFU)/mg of protein/min.

Multiplex ELISA: The commercially available Rat Inflammation ELISA Colorimetric Strip from Signosis (Santa Clara, CA, USA) was used to measure relative concentrations of tumour necrosis factor α (TNFα), interleukin 6 (IL6), interferon γ (IFNγ), interleukin 1α (IL1α), IL-1β, CCL2, CCL3 and CCL5 in tissue homogenates according to the manufacturer’s instructions. Relative concentrations of the analytes were expressed as optical density (OD) measured at λ_450nm_/mg of protein.

Wet-Weight to Dry Weight Ratios: The ratio of the tissue wet-weight to dry-weight (W/D ratio) was determined gravimetrically, as described [14].

Data Analyses and Statistics: Data are presented as mean ± standard error (SE). Data were analyzed with Fisher’s exact test, unpaired *t*-test, and two-way analysis of variance (ANOVA) with Tukey’s multiple comparisons test, as appropriate. All analyses were calculated with the GraphPad Prism programme version 10.3.1 (GraphPad Software, San Diego, CA, USA). A two-tailed *p* < 0.05 was considered significant.

## 3. Results

**Figure 1:**
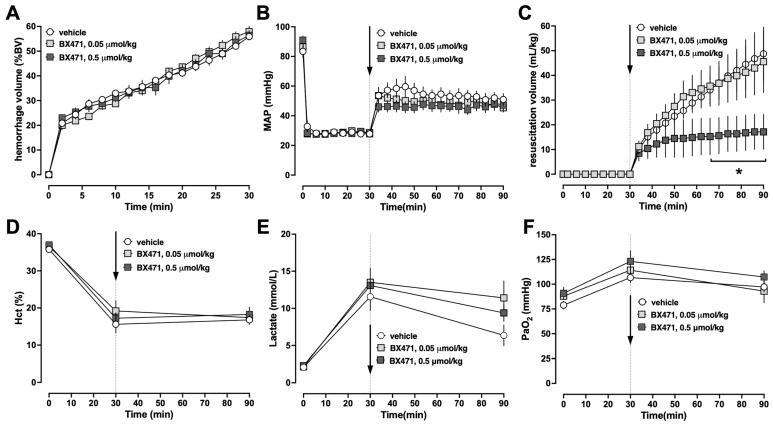
Animals were hemorrhaged to an MAP of 30 mmHg for 30 min, followed by fluid resuscitation for 60 min. At t = 30 min, animals were treated with vehicle (open circles, n = 5), 0.05 μmol/kg BX471 (light grey squares, n = 5), or 0.5 μmol/kg BX471 (dark grey squares, n = 4). Arrows indicate the time point of iv vehicle/drug injection. *: *p* < 0.05 vs. vehicle-treated animals. (**A**) hemorrhage volume in % of total blood volume (%BV). (**B**) MAP (mmHg). (**C**) Resuscitation volume (mL/kg). (**D**) Hematocrit (%). (**E**) Lactate concentrations (mmol/L). (**F**) PaO_2_, partial pressure of oxygen in arterial blood (mmHg).

To gain initial information on the effects of BX471 during resuscitation from hemorrhagic shock, animals were injected with a single intravenous (iv) bolus of vehicle, 0.05 μmol/kg or 0.5 μmol/kg BX471, at the end of the hemorrhage period, followed by crystalloid fluid resuscitation for 60 min (Series 1). There were no differences in any measured physiological parameters among groups at baseline. The hemorrhage volumes needed to achieve an MAP of 30 mmHg during the shock period were indistinguishable among groups, and all animals could be resuscitated to the target blood pressures (Figure 1A,B). While the fluid requirements to achieve blood pressure targets during the resuscitation period were indistinguishable between animals treated with vehicle or 0.05 μmol/kg BX471, treatment with 0.5 μmol/kg BX471 significantly reduced fluid requirements by more than 60% (Figure 1C). There were no significant differences in hematocrit values (Figure 1D), blood lactate concentrations (Figure 1E), or the partial pressures of oxygen in arterial blood between the groups (Figure 1F). All animals survived the observation period.

**Figure 2:**
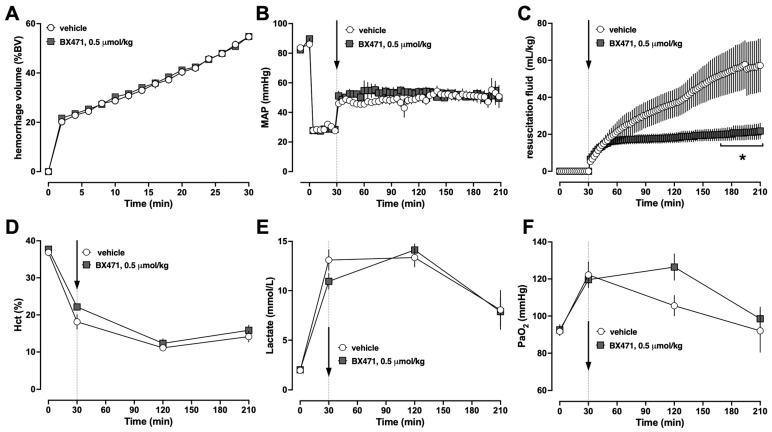
Animals were hemorrhaged to an MAP of 30 mmHg for 30 min, followed by fluid resuscitation for 180 min. At t = 30 min, animals were treated with vehicle (open circles, n = 8) or 0.5 μmol/kg BX471 (dark grey squares, n = 7). Arrows indicate the time point of iv vehicle/drug injection. *: *p* < 0.05 vs. vehicle-treated animals. (**A**) Hemorrhage volume in % of total blood volume (%BV). (**B**) MAP (mmHg). (**C**) Resuscitation volume (mL/kg). (**D**) Hematocrit (%). (**E**) Lactate concentrations (mmol/L). (**F**) PaO_2_, partial pressure of oxygen in arterial blood (mmHg).

Based on these findings, we selected a dose of 0.5 μmol/kg BX471 to assess whether the observed fluid-sparing effects would persist over a resuscitation period of 3 h (Series 2). To adhere to the 3Rs principles in animal experimentation, we omitted another treatment group that receives 0.05 μmol/kg BX471 in series 2 because this dose did not show any effects in series 1 [22]. As in series 1, the physiological parameters between vehicle and BX471-treated animals at baseline were indistinguishable and the hemorrhage volumes required to achieve an MAP of 30 mmHg were comparable (Figure 2A,B). When compared with vehicle-treated animals, treatment with BX471 significantly reduced the fluid requirements to maintain MAP targets by more than 60% (Figure 2C). Hematocrit values (Figure 2D), lactate concentrations (Figure 2E), and partial pressures of oxygen in arterial blood were indistinguishable between the groups (Figure 2F). Seven of the eight vehicle-treated animals and all BX471-treated animals survived the observation period (*p* > 0.05).

**Figure 3:**
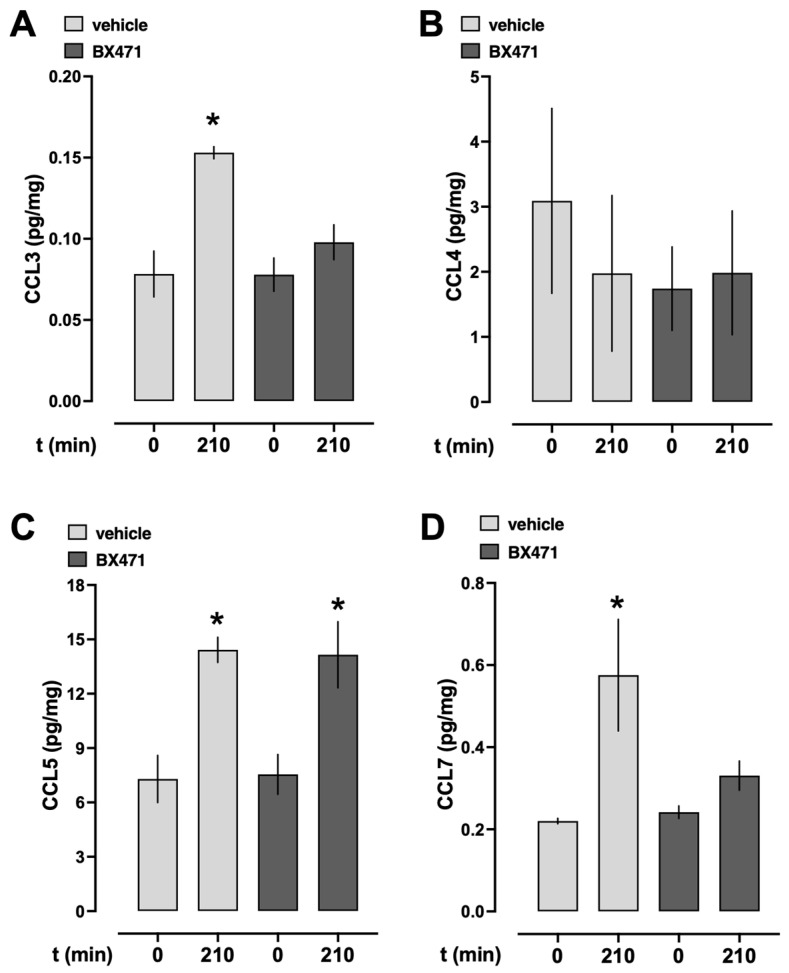
Serum CCR1 ligand concentrations (pg/mg) at baseline (t = 0 min) and at the end of the experiment (t = 210 min). Light grey bars: vehicle treatment, n = 7. Dark grey bars: 0.5 μmol/kg BX471 treatment, n = 7. (**A**) CCL3, (**B**) CCL4, (**C**) CCL5, and (**D**) CCL7. *: *p* < 0.05 vs. baseline (t = 0 min).

To assess whether endogenous CCR1 ligands were systemically released during resuscitation from hemorrhagic shock under our experimental conditions, we measured the serum levels of CCL3, CCL4, CCL5, and CCL7 at baseline and at the conclusion of the experiment (Figure 3). In vehicle-treated animals, CCL3, CCL5, and CCL7 serum levels significantly increased from baseline to the end of the experiment (Figure 3A,C,D), whereas CCL4 serum levels were not significantly altered (Figure 3B). While CCL5 serum concentrations also significantly increased from baseline to the end of the experiment in BX471-treated animals (Figure 3C), CCL3, CCL4, and CCL7 concentrations remained unchanged after BX471 treatment.

**Figure 4:**
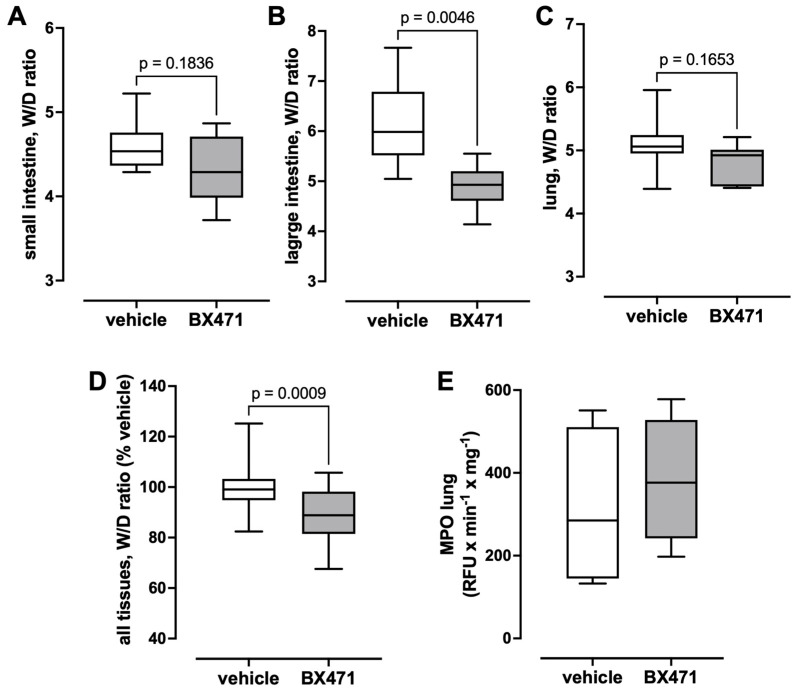
(**A**–**C**) W/D ratios of small intestine (**A**), large intestine (**B**), and lung (**C**). (**D**) W/D ratios of the sum of all tissues in % of vehicle. (**E**) Lung extract MPO activity (RFU/min/mg). Boxes extend from the 25th to the 75th percentile, and the horizontal line shows the median. Error bars show the range of data (minimum/maximum). Open boxes: vehicle treatment, n = 8. Grey boxes: 0.5 μmol/kg BX471 treatment, n = 7. The level of statistical significance is indicated.

The W/D ratios measured in small and large intestine and in the lung are shown in Figure 4A–D. Although the W/D ratios of the small intestine (Figure 4A) and the lung (Figure 4C) were lower in BX471-treated animals than in vehicle-treated animals, these differences did not reach statistical significance. BX471-treatment, however, significantly reduced the W/D ratios of the large intestine (vehicle—6.12 ± 0.29, BX471—4.9 ± 0.16, *p* < 0.05, Figure 4B). When the relative changes in the W/D ratios of the sum of all tissues (vehicle group = 100%) were calculated as a parameter of the global tissue effects of the treatment, the W/D ratio was 89.5 ± 2.2% with BX471 treatment (vehicle: 100 ± 2.2%, *p* = 0.0009 vs. BX471, Figure 4D). Furthermore, there were no significant differences in MPO activity in lung extracts between the groups (Figure 4E).

**Figure 5:**
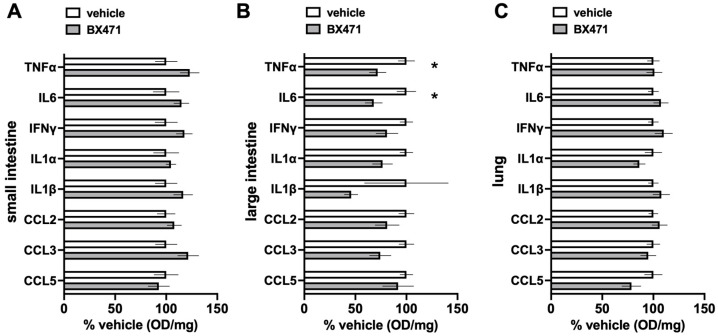
Inflammation markers in small intestine (**A**), large intestine (**B**), and lung (**C**) tissue extracts, as indicated in the graphs. Data are relative concentrations in % of the OD/mg measured in tissue extracts from vehicle-treated animals. Open bars: vehicle, n = 6. Grey bars: 0.5 μmol/kg BX471, n = 6. *: *p* < 0.05 vs. vehicle.

Figure 5 show the comparisons of the relative concentrations of a panel of inflammation markers in the tissue extracts. While we did not observe significant differences for any of the inflammation markers in extracts from the small intestine and the lung (Figure 5A,C), the relative concentrations of TNFα and IL-6 in large intestine extracts were significantly lower with BX471 treatment as compared with vehicle treatment (Figure 5B).

## 4. Discussion

In the present study, we show that the iv administration of BX471 at the beginning of fluid resuscitation from hemorrhagic shock significantly reduces the fluid requirements to stabilize hemodynamics in rat models. These fluid-sparing effects occurred dose-dependently and were not associated with noticeable adverse events.

As indicated by the consistent increase in lactate concentrations to 10–15 mmol/L at the end of the hemorrhage period and the development of significant fluid requirements to maintain hemodynamics, the hemorrhagic shock and resuscitation model that we employed in the present and in previous studies has reproducible pathophysiological consequences and thus can be used to place our current findings in the context of our previous observations [14,15].

Our experimental design addresses early resuscitation strategies, i.e., a scenario comparable to pre-hospital and emergency room resuscitation in patients. Although prehospital blood transfusion protocols have gained attention in recent years, most trauma patients in the US do not receive blood products in the pre-hospital setting. Moreover, a recent multicenter, allocation concealed, open-label, parallel group, randomized, controlled phase 3 trial did not provide evidence that very early blood product transfusion provides a benefit over crystalloid transfusion in trauma patients [23]. As such, our experimental design reflects common clinical practice and adherence to evidence-based medicine.

Our findings indicate that systemic concentrations of CCL3, an agonist at CCR1 and CCR5, CCL5, an agonist at CCR1 and CCR3, as well as CCL7, which activates CCR1, CCR2, CCR3, and CCR5, increase in vehicle-treated animals under our experimental conditions [2,16]. This implies that the release of endogenous CCR1 agonists during hemorrhagic shock and resuscitation contributes to increased systemic fluid requirements and provides a rationale for the beneficial effects of BX471.

The fluid-sparing effects of BX471 in the present study mimic our previous observations on the effects of the CCR2 antagonist INCB3284 and the CCR3 antagonist SB328437 in the same animal model [14,15]. This suggests that the activation of CCR1, CCR2, and CCR3 result in similar pathophysiological consequences after hemorrhagic shock and fluid resuscitation and implies at least some degree of redundancy regarding their effects on cardiovascular function. While serum concentrations of CCL5 increased in BX471-treated animals to a degree observed in vehicle-treated animals, significant increases in CCL3 and CCL7 concentrations could not be detected after BX471 treatment. This finding could be explained with a feed-forward mechanism, by which CCL5, via activation of CCR1, amplifies the acute inflammatory response and induces the systemic release of CCL3 and CCL7. This assumption would be consistent with the previously described effects of CCR1 knockout on the expression of CCL3 after renal ischemia–reperfusion injury [24]. The finding that CCL5 concentrations were not affected by BX471 treatment is in agreement with our previous findings, which showed that CCL5 concentrations are already significantly increased at time points before BX471 was administered in the present study [14].

Our observations in the present study that hematocrit values were comparable among groups and that lactate concentrations declined to a similar degree in vehicle and BX471-treated animals indicate that all animals were resuscitated to a comparable intravascular volume status and exclude the possibility that the fluid-sparing effects of BX471 are due to under-resuscitation. While BX471-treatment significantly reduced the W/D ratios of the large intestine, the W/D ratios of the small intestine and the lung were lower with BX471 treatment, but these differences did not reach a level of statistical significance. Because the W/D ratios of the sum of all tissues that were evaluated were also significantly reduced with BX471 treatment, our observations point toward the systemic reduction in the third-spacing of fluids as a possible mechanism through which BX471 reduced fluid requirements, rather than selective effects of BX471 in the large intestine.

The findings that there were no significant differences in the concentrations of a panel of inflammation markers in the small intestine and lung extracts from vehicle-treated and BX471-treated animals match our observations regarding the W/D ratios in these tissues. In contrast, TNFα and IL6 concentrations in large intestine extracts were significantly reduced after BX471 treatment as compared with large intestine extracts from vehicle-treated animals. While these single time point measurements should be interpreted with caution, they may suggest that CCR1 blockade exerted anti-inflammatory effects, which prevented increases in W/D ratios to the level observed in vehicle treated animals. Detailed time-course measurements of tissue W/D ratios and of inflammation markers in tissue extracts will be required to establish such correlations in the future.

There are several limitations of this pilot study. Although we did not observe adverse effects of BX471-treatment, we did not systematically monitor routine laboratory parameters that may reflect organ injury. Nevertheless, BX471 has already been used in clinical trials, and thus demonstrates a safety and toxicity profile that permits its use in humans [25]. Furthermore, this study is limited by its resuscitation period of 3 hrs. While this resuscitation period reflects pre-hospital and emergency room treatment periods in patients, we cannot address the effects of BX471 treatment on the development of secondary organ injury, such as lung injury or acute respiratory distress syndrome, which usually develop at later time points after hemorrhagic shock and fluid resuscitation. Similarly, we cannot comment on the possible effects of BX471 on survival because our model did not result in significant mortality within the observation period. In addition, while this study was designed to provide initial evidence for therapeutic efficacy of CCR1 blockade after hemorrhagic shock, it is unable to address the mechanisms by which these effects occurred. Nonetheless, because our observations suggest a reduction in third-spacing as a contributing mechanism, future studies on the roles of CCR1 in the regulation of endothelial permeability during hemorrhage and resuscitation appear warranted.

In conclusion, our findings suggest that CCR1 could be another new therapeutic target to reduce fluid requirements during resuscitation from hemorrhagic shock. Our findings justify further evaluation of the dose–effect profile and therapeutic potential of BX471 during longer resuscitation periods and in traumatic–hemorrhagic shock models that are associated with the development of organ injury and mortality. Because BX471 has already been evaluated in clinical trials, further evidence of the therapeutic potential of BX471 in preclinical models may facilitate clinical trials to evaluate its possible fluid-sparing properties after traumatic–hemorrhagic shock or in other conditions in which fluid overload is a significant concern.

## Data Availability

The original contributions presented in the study are included in the article; further inquiries can be directed to the corresponding author.

## Abbreviations

The following abbreviations are used in this manuscript:

ANOVA	Analysis of variance
BV	Total blood volume
CCL	Chemokine (C-C motif) ligand
CCR	Chemokine (C-C motif) receptor
ELISA	Enzyme-linked immunosorbent assay
Hct	Hematocrit
IFN	Interferon
IL	Interleukin
iv	Intravenous
LR	Lactated Ringer’s solution
MAP	Mean arterial blood pressure
MPO	Myeloperoxidase
OD	Optical density
PBS	Phosphate-Buffered Saline
SE	Standard error
TNF	Tumour necrosis factor
W/D ratio	Wet-weight to dry-weight ratio
